# Inhalation and inflammation: examining aeroallergens and eosinophilic esophagitis

**DOI:** 10.3389/falgy.2025.1609120

**Published:** 2025-06-16

**Authors:** Sitharthan Sekar, Kate Kelly, Dipa Sheth, Marianna Papademetriou

**Affiliations:** ^1^ Division of Gastroenterology, MedStar Georgetown University Hospital, Washington, DC, United States; ^2^ Division of Allergy and Immunology, George Washington University, Hospital, Washington, DC, United States; ^3^ Washington DC VA Medical Center, Washington, DC, United States

**Keywords:** eosinophilic esophagitis, allergens, pollen, seasonal allergies, aeroallergens, endotypes

## Abstract

Eosinophilic esophagitis (EoE) is a chronic immune-mediated disease leading to inflammation in the esophageal lining. EoE has become a significant cause of gastrointestinal illness in both children and adults. While there is significant focus on dietary triggers in the pathophysiology of the disease, aeroallergens are also increasingly implicated in both the development and clinical presentation. Possible mechanisms, seasonality and current evidence for the role of aeroallergens in EoE are discussed, including seasonality, allergen specific mechanisms and therapeutic options. A multidisciplinary team between allergists and gastroenterologists is optimal for coordinated patient management.

## Introduction

Eosinophilic esophagitis (EoE) is a chronic immune-mediated condition leading to inflammation in the esophageal lining. EoE has become a significant cause of gastrointestinal illness in both children and adults (1). The disease is characterized by the presence of eosinophils in the esophageal mucosa, leading to symptoms like dysphagia and food impaction. While there is significant focus on dietary triggers in the pathophysiology of the disease, aeroallergens are also increasingly implicated in both the development and clinical presentation. This review addresses the current understanding of EoE, covering its pathophysiology, clinical presentation with a focus on seasonality, allergen-specific mechanisms, and therapeutic options.

### Epidemiology

Prevalence and incidence of EoE have increased in recent decades, due to heightened awareness and increased availability of endoscopy. It has become one of the most common causes of dysphagia in much of the world (2). EoE frequently exists as part of an allergic disease spectrum. Many patients with EoE also have other allergic conditions such as asthma, allergic rhinitis, and eczema. The association supports the perception that EoE is a manifestation of systemic allergic hypersensitivity. Understanding the links between EoE and other atopic disorders may provide insights into shared mechanisms and inform treatment strategies. Allergens, in particular, play a significant role in the pathogenesis of EoE, as they can trigger immune responses that lead to the recruitment of eosinophils and other inflammatory cells to the esophagus (3–5).

### Immune mechanisms in EoE

The development of EoE is influenced by genetic factors, environmental triggers, and immune system activation, primarily involving type 2 T helper (Th2) cells (6). A key predisposing factor is the breakdown of the esophageal epithelial barrier, which allows dietary and aero allergens to enter the esophageal mucosa and trigger an immune response (7). The esophageal mucosa of patients with EoE exhibits high levels of thymic stromal lymphopoietin (TSLP), which connects innate and adaptive Th2-type immune reactions (8). This results in the release of cytokines like Interleukin (IL) IL-4, IL-5, and IL-13, needed for eosinophil activation and recruitment.

Chronic inflammation in EoE leads to tissue remodeling, fibrosis, epithelial thickening, and smooth muscle hyperplasia (9). Environmental triggers play a significant role in EoE, especially in those with other allergic conditions such as asthma, allergic rhinitis, and eczema (10). Aeroallergens can trigger similar immune responses that lead to the recruitment of eosinophils to the esophagus (11). Thus highlighting the importance of identifying and managing exposure to these specific allergens in patients with EoE.

New research further suggests the role of the esophageal microbiota in EoE and that changes in the esophageal microbiome may affect the course of the disease through immunomodulation and inflammatory regulation (12). Understanding the role of allergens in EoE is important for the future development of directed therapy and improved patient outcomes.

### Clinical features

EoE is associated with a variety of symptoms that can significantly impact quality of life. It is characterized by clinical symptoms of esophageal dysfunction and histologic evidence of eosinophil-predominant inflammation. Symptoms vary with age—adults frequently experience dysphagia, food impaction, and chest pain, while children can present with feeding issues, vomiting, abdominal pain, and failure to thrive (13). Patients can also endorse chest pain that mimics heartburn, but does not respond to proton pump inhibitors (14).

On initial evaluation, a detailed history is helpful to identify any potential allergic triggers of symptoms, including food and environmental allergens. Many patients with EoE have a history of atopic conditions such as asthma, allergic rhinitis, or eczema, which can help guide diagnosis (10).

When considering EoE other causes of esophageal eosinophilia must be ruled out. Gastroesophageal reflus disease (GERD), which can independently lead to esophageal eosinophilia, but also coexist with EoE. Rarer causes, such as parasitic infection, allergic vasculitis, esophageal leiomyomatosis, and Crohn's disease of the esophagus, should also remain on the differential (14).

Endoscopic examination is a valuable diagnostic tool for EoE. Linear furrows, white plaques, concentric rings, and strictures are common findings seen on examination, although some may have a normal appearance of their esophagus, making biopsy essential for diagnosis. Histologic features supporting EoE include basal zone hyperplasia, eosinophil degranulation, and dilated intercellular spaces, which aid in the overall diagnosis (15).

### EoE endotypes

Disease severity and progression are difficult to predict in individuals with EoE. By analyzing gene expression profiles, histologic features and endoscopic findings, researchers have described three distinct endotypes of EoE. Although the classification of endotypes is not yet routinely used in clinical practice, it's useful to understand variations in disease presentations. The first is EoEe1, which presents with an endoscopically normal-appearing esophagus and less severe histologic changes. The prognosis in EoEe1 patients appears favorable, and the disease is responsive to standard therapy like PPIs and topical steroids and is the least likely to develop into complex disease (16).

The second endotype, EoEe2, correlated with a strong type 2 inflammatory response that is not typically responsive to topical steroids. Patients have a worse prognosis and typically require alternative therapy in the form of biologics. However, with escalating therapy they can achieve symptom and histologic remission, although there is an increased risk of chronic inflammation (16). Both EoEe1 and 2 were associated with pediatric onset of disease.

The third phenotype, EoEe3 is associated with adult-onset disease and is most likely to be associated with narrow caliber esophagus. These patients may also present with fibro-stenotic features like strictures. This phenotype is associated with severe genetic changes and the least expression of epithelial differentiation genes. These patients often need the combination of medical therapy and endoscopic therapies like dilation to manage strictures. Long-term management is necessary to prevent further complications and maintain quality of life (16, 17).

The primary differences between the endotypes was degree of histological change with focus on degree of basal zone hyperplasia and eosinophilic involvement, lowest for EoEe1 and highest for EoEe3. See Table 1 for a comparison between EoE endotypes (16). Notably, the role of aeroallergens in presentation, severity or exacerbations were not examined in the evaluation of the proposed endotypes.

**Table 1:** Comparison of characteristics of proposed Eosinophilic Esophagitis endotypes.

Feature	EoE1	EoE2	EoE3
Histologic severity	Mild	Moderate	Most severe
Basal zone hyperplasia	Minimal	Significant	Extensive
Endoscopic appearance	Normal	Classic EoE changes including distal furrows	Narrow caliber
Gene expression	None prominent	High inflammatory cytokines and steroid-response genes	Low expression of epithelial differentiation genes
Clinical associations	Unlikely to require esophageal dilation	Inflammatory phenotype	Adult onset, often fibrostenotic disease

### Aeroallergens and EoE

**Figure 1:**
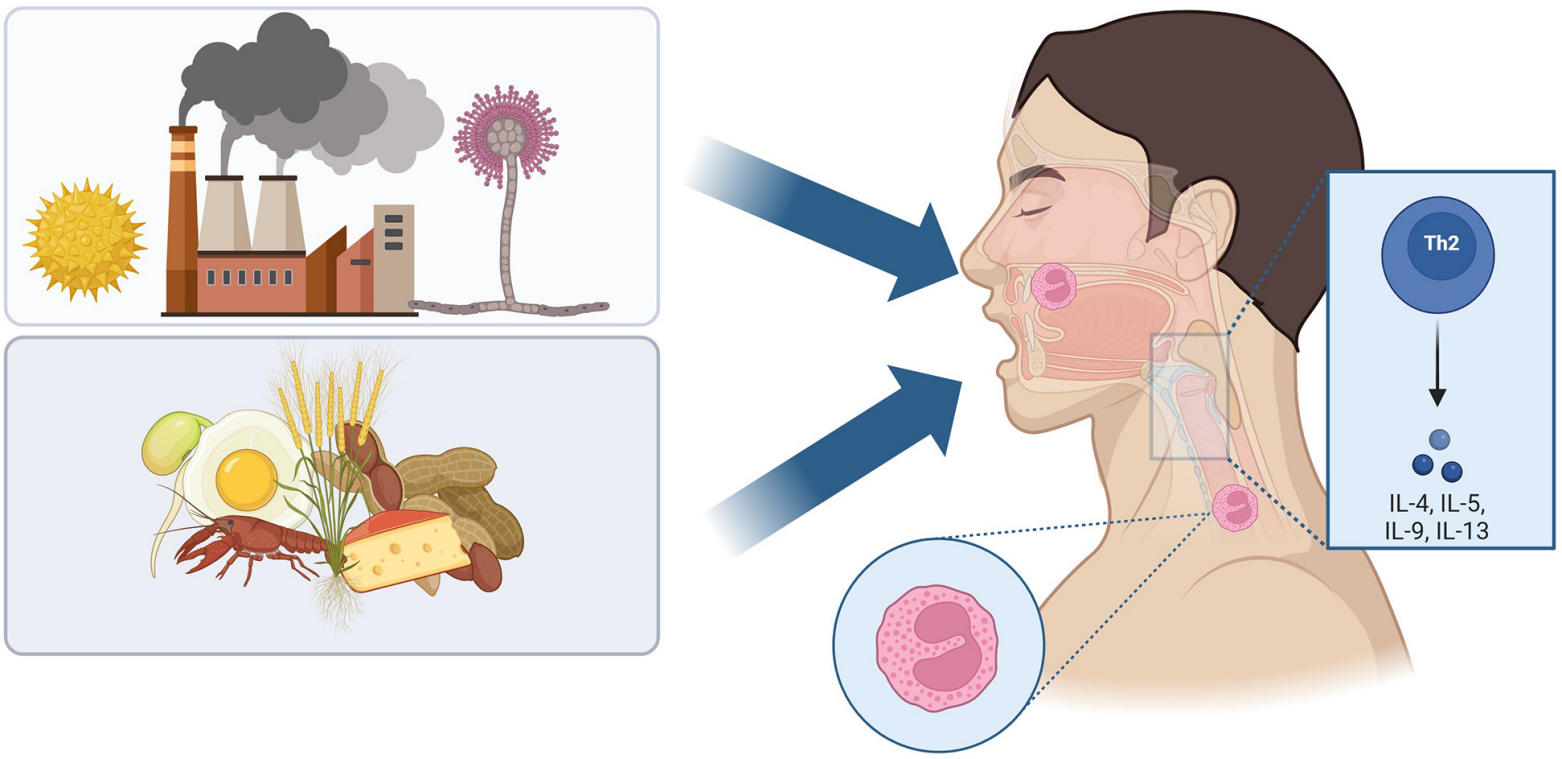
Aeroallergen and dietary allergen exposure contribute to the immune response of Eosinophilic Esophagitis. Created in biorender.com.

EoE often coexists with other atopic diseases like asthma, allergic rhinitis, and atopic dermatitis. Food allergens are thought to be the main driver in the development of the disease. Moreover avoidance of food allergens is a mainstay of long term therapy. However, there is increasing interest in aeroallergens and the incidence of EoE (18) (Figure 1).

Studies done in mice without eosinophilic esophagitis have developed symptoms of the disease after exposure to a known aeroallergen (19). In one study, mice were exposed to intranasal aspergillus fumigatus allergens and developed the same presentation of EoE as found in humans. Interestingly, when exposed to the same allergens via oral or gastric route, these mice did not present with EoE, suggesting a correlation and similar mechanism of hypersensitivity in the respiratory tract and the esophagus. In the same study, perennial, year round allergens—specifically dust mite and cockroach—found in many family homes were also introduced nasally into mice. These mice were later found to have increased eosinophils and mast cells in the esophagus. Humans sensitized to similar year round aeroallergens, especially cockroaches and dust mites, are found to be less responsive to traditional management of EoE that entails diet management and steroids (19).

While most studies conducted on EoE note the presence and activity of eosinophils, few note the presence of specific allergens. One study biopsied the esophagus of 129 patients with EoE, 82 of whom reported having seasonal exacerbations. They used sirofluor fluorochrome to stain for callose, a substance found abundantly in pollen but normally absent in human tissue. Biopsies of the patient's esophagus were taken during periods of active EoE, most of which occurred during pollen season. Sixty-seven percent of the biopsies detected callosa in the esophagus compared to none in the controls. The biopsies showed pollen tubes in the esophagus surrounded by eosinophils contributing to microabscesses that are often seen on diagnostic biopsies (20).

While aeroallergen exposure has not been fully implicated in the original pathogenesis of EoE, studies like these suggest that one's local environment and exposure to aeroallergens may influence disease burden and responsiveness to treatment.

### Seasonality

Aeroallergen levels are associated with changing seasons and climate. For geographic areas that experience temperature change and seasons, there are a variety of aeroallergens that may predispose patients to EoE. Perennial allergens are mold, dust mites, and pet dander. Tree pollen is implicated in the spring, grass pollen in the summer, and weed pollen implicated most in the fall. The seasonality of these allergens in addition to the demonstrated link between aeroallergens and EoE has the potential to impact both the times in which EoE presents and is diagnosed as well as when exacerbations may occur.

Patterns have been noted in the seasons in which most EoE is diagnosed. Studies found that EoE in children was diagnosed far less frequently in the winter time. It was shown by Wang et al. in their retrospective study that only 14% of new diagnoses of EoE were during winter time, as compared to 45% in spring, 19% in summer, and 22% in fall. In addition, the severity of inflammation found in newly diagnosed patients was less in patients diagnosed in winter months (21).

A single center study in the south central region of the United States noted seasonal variation in the presentation of EoE in children, with two peaks in diagnosis in the spring and the fall. 45% of cases were diagnosed in the spring and the next highest was 22% of cases diagnosed in the fall. These increases correlate with the region's peak levels of aeroallergens: where grass and tree pollen are at their highest in the spring and weed pollen in the fall (22). While more data is available for children, a seasonal prevalence of EoE in adults has also been found. A small consecutive one year retrospective study found that adults were more often diagnosed with EoE during the spring, with 28 v 13 new cases (*p* = 0.002) (23). The increased frequency of diagnoses during non-winter months when allergen levels are at their highest seem to suggest a role for aeroallergen in potentiating the disease.

Exacerbations of EoE, defined as increase in esophageal eosinophil count to greater than 15, have been linked to aeroallergen exposure. One case report from the northeast United States demonstrated exacerbation of symptoms and increased eosinophilic infiltration in the esophagus in a patient not on any therapy for EoE during predictable times of the year where pollen counts were high. Despite no changes in diet, biopsy specimens revealed a pattern of elevated eosinophils in the spring and summer months, while winter biopsies revealed only mild if any eosinophilic infiltrate histologically as well as corresponding improvement in symptoms (24).

A retrospective study completed at a children's hospital in the United States analyzed how disease course changed in children with biopsy-confirmed aeroallergen induced EoE during seasons. While those with confirmed aeroallergen induced EoE made up only 3% of the total participants, all of them experienced seasonal variation and flares, with at least a two fold increase in eosinophil count. Most notably, these flares occur most often in the spring and summer, calling into question the aeroallergens most prevalent at that time of year (24, 25). In addition, trials done in animal models have noted the onset of EoE was consistent with antigen inhalation (26). These findings in both animals and humans implicate aeroallergens in the presentation and severity of EoE, and therefore may be correlated to the season in which aeroallergens are the most prevalent.

A retrospective cohort study conducted in the mid-Atlantic region of the United States found similarly that while exacerbations of EoE were rare, patients who experienced them carried a history of other atopic conditions such as allergic rhinitis, IgE-mediated food allergy, and atopic dermatitis. Flares, defined as a doubling of eosinophil count in those in remission (less than 15 per hpf), were mostly experienced in the summer and fall (27). The seasonality of these flares, in combination with the atopic conditions and the seasons they present in may implicate grass or weeds and mold for occurrence of the summer and fall exacerbations, respectively.

Most research on EoE has been conducted within Western nations like the United States that experience variation in climate throughout the year. There is limited literature on the presentation and characteristics of EoE in other countries, especially in those that experience little to no change in season throughout the year. With the present research, it can be hypothesized that in regions of the world with more tropical climate and less distinct seasons, aeroallergens may be at consistent levels and non-contributors in the presentation and exacerbations of EoE. For this reason, future studies should be conducted to better understand the geographic distribution of EoE as this may help to correlate its prevalence and severity with seasonality.

Attempts to treat EoE by controlling concomitant allergies offers insight into the role seasonality plays in this disease. Different cases noted the effect that treating aeroallergens has on patients with EoE. In many of these cases, immunotherapy to seasonal allergens has shown some relief of symptoms and disease burden of EoE in these patients when traditional treatment for EoE was unsuccessful (28). Though multifactorial, successful treatment in these cases highlight the important role the environment plays in the pathogenesis of EoE.

### Allergen specific mechanisms

Local and systemic mechanisms may both promote development and exacerbations of EoE. On the local level, it is theorized that large amounts of inhaled pollen may introduce antigens into the esophagus. Furthermore, congested patients who tend to inhale more through their mouth offer a more continuous route for antigens to reach the upper parts of the alimentary tract. This antigen exposure then promotes IL-5 to recruit eosinophils to the esophagus with other inflammatory mediators, as previously described. Some data support the claim that the introduction of aeroallergen into the GI tract is responsible for worsening eosinophilia in the esophagus, but as mentioned above- respiratory exposure is related to the eosinophils found in the esophagus (19).

Another theory is that traditional food elimination used to treat EoE has not accounted for some foods with cross-reactivity to pollen (27). PFAS, or Pollen Food Allergy syndrome, is a reaction syndrome that takes place after the ingestion of food with an antigen that is highly homologous with pollen antigens that a patient has been previously sensitized to (29). This syndrome is described as a process in which mast cells bound with aero-allergen specific IgE antibodies degranulate in the presence of re-exposure to antigens.

One study investigating EoE found that eosinophilic infiltration of the esophagus was prominent in patients with respiratory pollen allergy. Researchers also noted that while not statistically significant, more infiltration was found in the proximal esophagus when compared to the distal esophagus- suggesting a route of exposure from the nasal passage into the upper GI tract. This idea further implicates the sequelae of allergic rhinitis exacerbated by exposure to allergens like pollen and finding requires further investigation to determine if treatment of allergic rhinitis impacts the presentation and severity of EoE (30).

Notably, an association between allergic rhinitis and EoE has been demonstrated due mechanisms in rhinitis that contribute to the introduction of aeroallergens into the esophagus (26). Due to the connection of allergic rhinitis and EoE symptoms, treatments aimed at treating allergic rhinitis, like sublingual immunotherapy (SLIT) and subcutaneous immunotherapy (SCIT), have been explored and their effect on EoE noted. SLIT is a form of immunotherapy that aims to switch the body's inflammatory response from domination by Th2 to TH1 cells in patients with allergic rhinitis or other allergies. Interestingly, case reports have demonstrated recurrence of EoE in patients undergoing SLIT, but an improvement and even remission in symptoms for those who underwent SCIT (26). More research is needed to fully understand this discrepancy but is a factor to consider when associating mechanisms of EoE introduced by allergens and its relation to allergic rhinitis.

### Treatment approaches for EoE

Care of EoE patients is optimal with a multidisciplinary approach between gastroenterology, allergists, registered dieticians, and pulmonologists depending on comorbid conditions. The management of EoE spans from dietary changes to medications, and sometimes repeated esophageal dilation. The first step is establishing, through shared decision-making, whether to focus on dietary or pharmacological treatments.

Elimination diets are one of the potential cornerstones of EoE management, with varying success rates in adult and pediatric populations. Elemental diets remain most effective particularly in pediatric populations. Empiric exclusion diets offer compromise between efficacy and practicality with recent data demonstrating high success rate with single food elimination (31). Limitations include long term maintenance on restricted diets, the possibility of multiple culprit allergens in the same individual, and the need for frequent upper endoscopies for histologic evaluation during the elimination and reintroduction phases.

Pharmacologic therapy for EoE includes several effective options, with ongoing research into new treatments. Proton pump inhibitors are most commonly the initial treatment due to their safety and ease of administration, with an approximately 41% histologic response rate (1). Topically ingested corticosteroids, like budesonide and fluticasone, are highly effective, with approximately 60% rates of histologic remission (1). Biologics like dupilumab, which inhibit IL-4 and IL-13, have shown significant improvements in clinical symptoms, histologic inflammation, and endoscopic scores, and therefore are a promising option for treatment refractory patients (32). For patients with multiple coexisting atopic conditions, dupilumab may be an appropriate first line treatment.

Newer promising, therapies for EoE include Etrasimod, a selective sphingosine 1-phosphate receptor (S1PR) modulator, and Janus kinase-signal transducer and activator of transcription (JAK-STAT) pathway inhibitors with potential due to its immunomodulating effects to help in those whose mechanism is via aeroallergens (33, 34).

## Conclusion

In conclusion, aeroallergens may play a significant role in both the development and exacerbations of EoE. The novel role of aeroallergens in the pathogenesis of EoE makes our understanding and treatment of EoE more complex. Aeroallergens can potentially elicit immune responses that form the basis for eosinophilic inflammation of the esophagus. As we further evolve our understanding and treatment of EoE, it is imperative that we further the relationship between gastroenterologists and allergists. Through collaboration, we can increase diagnostic accuracy, develop new therapies, and enhance patient education and engagement. As the field continues to grow, longitudinal, large scale studies would help solidify the relationship between aeroallergens and EoE. The future of EoE management lies in our ability to integrate multidisciplinary perspectives and address the diverse factors contributing to this challenging disease.
